# Point Mutations in Furazolidone and Rifampicin Resistance Genes in *Helicobacter pylori* Strains from Colombia

**DOI:** 10.3390/antibiotics13070643

**Published:** 2024-07-12

**Authors:** Kevin Andres Guzman, Arsenio Hidalgo, Alvaro Jairo Pazos

**Affiliations:** 1 Grupo Salud Pública, Centro de Estudios en Salud de la Universidad de Nariño (CESUN), Universidad de Nariño, Pasto 520001, Colombia; archi@udenar.edu.co (A.H.); alpazmo@udenar.edu.co (A.J.P.); 2 Departamento de Matemáticas y Estadística, Facultad de Ciencias Exactas y Naturales, Universidad de Nariño, Pasto 520001, Colombia; 3 Departamento de Biología, Facultad de Ciencias Exactas y Naturales, Universidad de Nariño, Pasto 520001, Colombia

**Keywords:** *Helicobacter pylori*, antibiotic resistance, gastric cancer

## Abstract

The eradication of *Helicobacter pylori* is a valid strategy for preventing gastric cancer; however, the therapeutic failure of first-line treatments in Colombia is associated with high resistance to metronidazole and amoxicillin. This study explored alternative antibiotics and analyzed point mutations in resistance genes to furazolidone and rifampicin in order to include them in rescue therapy regimens. A total of 239 complete genomes of *Helicobacter pylori* Colombian strains were compared to that of the ATCC 26695 strain to identify mutations in the *rpoB* and *porD* genes for rifampicin and furazolidinone resistance, respectively. While rifampicin resistance mutations were not found, only 0.84% of the isolates showed the *porD* gene, suggesting that *Helicobacter pylori* is sensitive to these antibiotics. A phylogenomic analysis of *Helicobacter pylori* revealed an independent lineage in Colombia (hspColombia). The absence of point mutations in the *rpoB* gene, together with the scarce mutations identified in the *porD* gene of *Helicobacter pylori*, suggest that the hspColombia isolates are sensitive to rifampicin and furazolidone, which could be key to including these antibiotics in the rescue therapies against *Helicobacter pylori*.

## 1. Introduction

*Helicobacter pylori* (*H. pylori*) is a bacterium that triggers an inflammatory process, which begins with chronic gastritis and progresses to peptic ulcer, atrophic gastritis, intestinal metaplasia, gastric dysplasia, and adenocarcinoma or MALT lymphoma [1,2]. The natural history of this infection is closely connected to the pathogenesis of gastric cancer, beginning at childhood when it is acquired by vertical transmission (from parents to children) and persisting throughout the life span of the host if the infection is not properly treated [3]. Even though *H. pylori* is classified as a Type I carcinogen, the prevalence of the infection is not directly correlated with the incidence of gastric cancer. Indeed, the development of gastric lesions is associated with the type of strain, host genetic susceptibility, host-bacterium coevolution, diet, as well as socioeconomic and environmental conditions [4,5].

The incidence of gastric cancer varies between different geographic regions worldwide. For instance, the incidence of cancer in Latin America ranges from 20 to 30 cases per 100,000 inhabitants, being more frequent in mountainous areas and less common in coastal regions [6]. In Colombia, two regions within the Department of Nariño clearly show these marked differences in the risk of gastric cancer. In the Andean region of Tuquerres, the incidence of gastric cancer is among the highest in the country, affecting 150/100,000 inhabitants. In contrast, Tumaco, which is located on the Pacific coast, shows a relatively low incidence (6/100,000) [7]. Interestingly, both regions have a similarly high prevalence of *H. pylori* infection (~90%) [8].

The eradication of *H. pylori* is the current recognized and effective strategy to reduce the incidence of gastric cancer. In Colombia, the antimicrobial resistance rate to first-line treatments against *H. pylori* is rising. Bacterial resistance to metronidazole, levofloxacin and amoxicillin has increased [9]. Consequently, the search for alternative antibiotics, such as furazolidone and rifampicin, has been proposed to replace those showing high resistance rates.

Nevertheless, in Colombia there is a limited number of studies focused on the bacterial resistance to furazolidone and rifampicin due to their restricted use in *H. pylori* eradication treatment. Furthermore, *H. pylori* is a microorganism that is difficult to grow under laboratory conditions, and the antibiotic susceptibility tests require longer periods of time. Therefore, novel methods that identify mutations in SNPs associated with antibiotic resistance, such as qPCR and Next Generation Sequencing (NGS), have become more precise approaches to providing better treatment options to mitigate infections caused by *H. pylori*.

Furazolidone, one of the nitrofurans used in the treatment of *H. pylori* as a rescue therapy, acts as a bacteriostatic agent with a mechanism of action similar to nitroimidazoles. Resistance to this antibiotic is mediated by nitroreductases such as flavodoxin pyruvate oxidoreductase (encoded by the *porD* gene). Mutations linked to furazolidone resistance in the *porD* gene involve guanine-to-adenine transitions at position 353 (G353A), adenine-to-guanine at position 356 (A356G), and cytosine-to-thymine at position 357 (C357T) [10].

Rifampicin binds to the β subunit of the DNA-dependent RNA polymerase, which is encoded by the *rpoB* gene of *H. pylori*, forming a stable complex that disrupts RNA synthesis. Point mutations in the V149F, Q527R, D530, and H540 codons of the *rpoB* gene can confer resistance to rifamycins by reducing the subunit’s affinity [11].

The purpose of this study is to conduct genomic surveillance of circulating strains and determine the resistance rate to furazolidone and rifampicin in *H. pylori* in Colombia.

## 2. Results

The bioinformatics analysis of *H. pylori* sequences revealed that the 16 isolates showed, on average, 115 contigs. The genomes had sizes ranging from 1,595,752 bp to 1,700,565 bp, with a mean of 1,663,692 bp ± 7,365,295. The analysis revealed a mean G/C content of 39.1% ± 0.05, with minimum and maximum values of 38.8% and 39.5%, respectively. Furthermore, the average number of genes in *H. pylori* was 1640, fluctuating between 1561 and 1796 (Table 1).

Finally, a total of 239 *H. pylori* isolates were analyzed in this study, of which 173 came from the central and mountainous Andean region of Colombia, mainly from the city of Bogota. Likewise, 57 isolates came from the Department of Nariño, whereas 7 were from the Department of Tolima.

The analysis of point mutations in the *rpoB* gene for rifampicin resistance did not identify any mutation in the Colombian isolates. The similar analysis for furazolidone revealed two isolates (2/239) with the C357T mutation (0.84%).

In addition, the C357G mutation was observed in four isolates of *H. pylori*. Nevertheless, to date, and to the best of our knowledge, there are no published data regarding the association of this mutation with *H. pylori* resistance to furazolidone.

**Figure 1:**
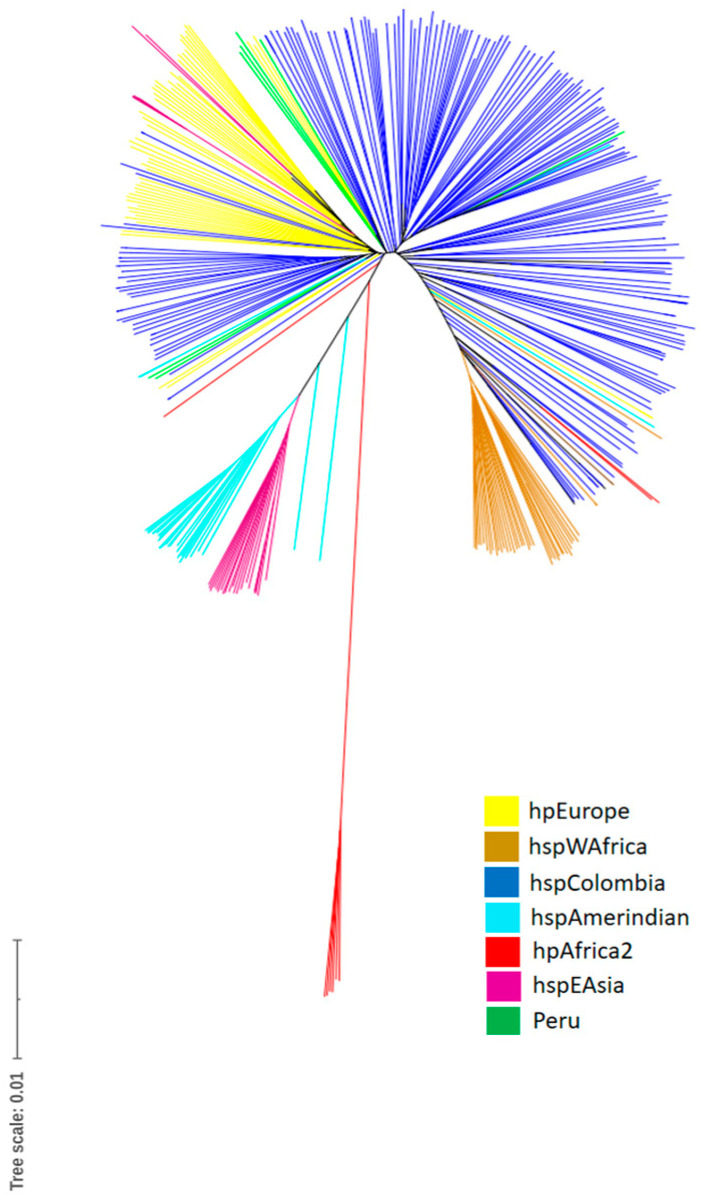
Phylogenomic tree based on the core genome of *Helicobacter pylori* isolates. The different populations (hp) and subpopulations are described on the right side of the phylogenetic tree.

The tree that originated from the phylogenomic analysis based on the core genome also shows the formation of independent clades of hpEurope, hspAmerindian, hspEAsia, hspWAfrica, and hspColombia, with hpAfrica2 being the most ancestral group. Some strains from the Pacific region of Colombia were included within a clade grouped with African strains (hpAfrica). The Colombian strains were also observed to be grouped with strains of European origin, which indicates that the horizontal exchange of genetic material between strains from geographically isolated populations appeared after the colonization of the Americas by the Spaniards (Figure 1).

## 3. Discussion

In Colombia, the arrival of Europeans to the American continent triggered the most notable miscegenation in human history, merging genetic pools from Africa, Europe, and America [12]. The Colombian Pacific coast shows a strong African influence in terms of both population ancestry and the *H. pylori* strains that infect these communities, which have ancestral similarities to bacterial variants found in Africa [13]. It is important to highlight that *H. pylori* has accompanied humanity since the dawn of *Homo sapiens* on the African plains, and this extended co-evolution period has resulted in a relatively low incidence of gastric cancer in the African populations. This phenomenon is known as the “African enigma” [14].

This enigma has been extrapolated to the Pacific coast of Nariño, Colombia, as one of the possible theoretical explanations for the low incidence of gastric cancer in this region [13]. Moreover, *H. pylori* strains from the Pacific have a higher resistance rate to first- and second-line antibiotics compared to strains found in the Andean region, which can be the result of a prolonged period of co-evolution of bacterium and host, as well as the frequent treatments for different local infections [4,13,15].

In contrast, the Colombian Andean region, where the incidence of gastric cancer is high, has an ancestral composition that originated the mixing between Europeans and Native American populations [13]. This mixture has given rise to a new environment for bacteria [16]. Specifically for *H. pylori*, the gastric niche present in these mestizo populations has triggered the appearance of a new strain known as hspColombia, which may have arisen from horizontal gene transfer between hpEurope and hspAmerindian, intragenomic recombination, new neo-tropical dietary habits, immune response, and/or the renewed gastric ecosystem [4,16]. All the aforementioned factors created a selective pressure on these bacterial communities.

Previous studies have reported that *H. pylori* from the Colombian Andean region has a *vacA* gene with a higher frequency of s1m1 alleles (Higher risk), whereas it is more common to find alleles s2m2 (Lower risk) in bacteria from the Pacific region of the country [17]. In addition, dietary factors may play an essential role in developing gastric cancer, as the Andean region consumes mainly foods with high carbohydrate content, while the Pacific coast diet is characterized by being rich in fruits with antioxidants, fresh vegetables, and seafood [18].

In Colombia, it has been observed that the presence of the 1082 (A/G) point mutation in the *IL-10* gene (79%), together with another mutation in position 511 of the *IL-1β* gene (72%) and the Th1 immune response, increase the risk for gastric cancer in human populations [4]. These findings highlight the complex interactions between genetic, environmental, and dietary factors that predispose the different regions of Colombia to developing gastric cancer.

This study focused on assessing *H. pylori* resistance to antimicrobial treatments based on furazolidone and rifampicin, with the purpose of exploring potential therapeutic alternatives. In terms of antibiotic resistance, the Colombian isolates did not exhibit mutations in the *rpoB* gene that were associated with rifampicin resistance, which suggests a potential effectiveness of this antibiotic in the treatment of *H. pylori* infections. This finding is consistent with a previous study carried out in Colombia that applied phenotypic techniques and DNA sequencing methods, reporting equally low resistance to furazolidone and rifampicin and supporting the results presented here [19].

In addition, mutations were identified in the *porD* gene that are associated with furazolidone resistance. Nevertheless, the incidence of resistant isolates reported in this study was minimum, representing only 0.84% of the total, which suggests that this antibiotic could be an excellent therapeutic alternative. It is important to highlight that a study about the phenotypic resistance to this antibiotic carried out in Bogota, Colombia, reported a resistance rate of 4.8% [10].

In Colombia, the treatment of *H. pylori* typically relies on a combination of amoxicillin, metronidazole, and clarithromycin as first-line therapy in conjunction with Proton Pump Inhibitors (PPIs). However, depending on the success or failure of empirical therapy, tetracycline or levofloxacin is used as second-line therapy, depending on the resistance rate of *H. pylori* to metronidazole or amoxicillin.

*H. pylori* resistance to first- and second-line antibiotics has increased in Colombia, which is demonstrated by the resistance rates being higher than the threshold established for an optimal treatment. Specifically, metronidazole, amoxicillin, and levofloxacin resistance rates have reached 93%, 25.9%, and 12.04%, respectively [9]. Given this scenario, and considering the high incidence and mortality of gastric cancer in the country, it is imperative to find new therapeutic strategies to eliminate *H. pylori* from gastric mucosa.

Given the increasing problem of *H. pylori* resistance, rifampicin and furazolidone have been proposed as alternatives for eradicating the bacteria. Under this scheme, a 14-day regimen has been suggested that includes the following components for furazolidone: PPIs, tetracycline, bismuth, and furazolidone. In the case of rifampicin, a similar 14-day regimen has been proposed that consists of PPIs, amoxicillin, bismuth, and rifampicin [20].

*H. pylori* is a genetically diverse microorganism, and treatment regimens often fail due to human genetic variability, stomach physiology (such as pH), and *H. pylori*’s own evolutionary lineage; therefore, it is essential to conduct further phenotypic and genotypic studies on clinical strains. These studies will help understand the most optimal treatment. Additionally, it is crucial to integrate susceptibility testing alongside empirical regimens in the bacteria eradication protocol [20].

This study provides a fundamental approach that can be applied in other countries in the region or anywhere in the world. The use of Next Generation Sequencing techniques in combination with genomic analysis makes it possible to carry out accurate diagnoses and determine the molecular epidemiological status, including the antimicrobial resistance of pathogens relevant to public health [9].

In the future, real time PCR commercial kits could be developed for *rpoB* and *porD* genes, which could be used to identify phenotypic resistance using samples obtained from gastric biopsy tissues of fecal samples [10,21]. Given the difficulties of growing *H. pylori* in vitro and the prolonged incubation times, this diagnosis and treatment strategy could represent a novel and efficient alternative in the fight against this microorganism in Colombia.

Nevertheless, a limitation of our study lies in the possibility that there are other genes with multiple mutation sites that could also affect antibiotic resistance levels. These mutations have not yet been identified by phenotypic or molecular testing, which suggests the need for additional research in this area to fully understand the antimicrobial resistance landscape in *H. pylori*.

## 4. Materials and Methods

### 4.1. Samples Provided by This Study

The 16 whole genome sequences correspond to *H. pylori* isolates collected between 2017 and 2022 in Nariño, Colombia. All participants signed the informed consent. The average age of patients was 52 years old, with a range from 30 to 68 years old. Regarding the gender of participants, 3 were male and 13 were female. Based on the histopathology results, 3 patients showed active chronic gastritis, 8 atrophic gastritis, and 5 intestinal metaplasia (Table 2).

### 4.2. DNA Extraction and Bioinformatics Analysis

*H. pylori* was grown in Petri dishes containing Columbia agar (Oxoid, Basingstoke, UK) supplemented with 10% defibrinated lamb blood, Dent selective supplement (Oxoid, UK), and 1% Isovitalex enrichment supplement (Oxoid, UK). Plates were incubated at 10% CO_2_ and 37 °C for 7–10 days. DNA extraction was carried out using the QIAmp DNA Mini kit (QIAGEN, Hilden, Germany). The MiSeq TM platform (Illumina, San Diego, CA, USA) was used for whole genome sequencing. The library was prepared through the Nextera XT (Illumina) kit, followed by 2 × 300 bp paired ends, which generated a 40X coverage. The FastQC v 0.12 software was used for quality control of the raw sequencing data [22]. The Trim Galore v 6.6 and Cutadapt v 4.3 programs were used to clean the data and to ensure its integrity and accuracy [23,24]. The de novo genome assembly was achieved through the SPAdes v 13.3 program [25]. Finally, the genomes were annotated at the NCBI database using the Prokaryotic Genome Annotation Pipeline (PGAP) [26]. All *H. pylori* complete genome sequences are available at NCBI (BioProject: PRJNA984677).

### 4.3. Antibiotic Resistance Mutations

An analysis of *H. pylori* genomes was carried out using data from Colombia that are available on the PubMLST platform (https://pubmlst.org/), which keeps relevant genetic information of several bacterial strains. We accessed 223 *H. pylori* specific genomes from Colombian strains registered at PubMLST (https://pubmlst.org/) together with the 16 isolates that were sequenced by our research group. Ultimately, the 16 genomes sequenced for this study, combined with the 223 genomes obtained from the database, totaled 239 genomes for Colombia in this analysis.

We used the 26695 reference strain as a starting point to investigate mutations in the *H. pylori* isolates. The proteins for mutation analysis were obtained from the reference strain 26695. The protein sequence (Ref: A0A2I8V4C0) was used for the *rpoB* gene, and the protein (Ref: O25737) was used for the *porD* gene, retrieved from UniProt (https://www.uniprot.org/). We studied the *rpoB* gene, as it has been associated with rifampicin resistance. Specifically, we assessed mutations in positions V149F, Q527R, D530, and H540. Meanwhile, we analyzed positions G353A, A356G, and C357T of the *porD* gene because of their association with furazolidone resistance (Table 3). 

### 4.4. Phylogenomic Analysis

The genome sequences of *H. pylori* isolates were obtained from BIGSdb (Bacteria Isolate Genome Sequence Database) [30]. Subsequently, a gene-by-gene alignment was carried out using DCSs (DNA Coding Sequences) of the J99 *H. pylori* reference strain (ID: NZ_JABFHN010000001.1). The resulting genome comparison matrix, containing 1694 genes of the *H. pylori* core genome, was used to construct a phylogenomic tree through the MEGA X software v. 10 [31] with 1000 bootstrap replicates. The phylogenomic tree was visualized and edited using iTOL v5 [32].

## 5. Conclusions

The absence of point mutations in the *rpoB* gene and the low number of mutations in the *porD* gene of *H. pylori* suggest that the Colombian isolates (hspColombia) are sensitive to rifampicin and furazolidone. This low resistance rate is a strong evidence to support the inclusion of furazolidone and rifampicin in rescue therapies against *H. pylori* in Colombia. Given that a *H. pylori* lineage (hspColombia) was found to be specific to Colombia, the resistance exhibited against antimicrobials is related to the local regimen administered in the country. This finding could provide essential information to design better treatment strategies against the hspColombia strain in the future.

## Figures and Tables

**Table 1:** Summary of reported *Helicobacter pylori* genome sequences.

Strain Name	GenBank Accession No.	Size Genome	N50 *	GC%	No. Contigs	Annotated Genes
AP002	JAUQTH000000000	1,668,722	31,913	39.22	157	1649
AP015	JAUQTI000000000	1,689,940	47,025	39.23	185	1692
AP018	JAUQTJ000000000	1,700,565	36,365	39.21	143	1678
AP021	JAUQTK000000000	16,80,516	63,185	39.22	167	1674
AP022	JAUQTL000000000	1,638,615	107,077	38.86	52	1595
AP025	JAUQTM000000000	1,652,684	60,512	38.95	66	1609
AP028	JAUQTN000000000	1,673,711	38,220	39.20	167	1685
AP031	JAUQTO000000000	1,653,928	10,422	39.50	46	1586
CR004	JAUQTP000000000	1,680,991	87,648	38.95	90	1627
CR005	JAUQTQ000000000	1,649,918	73,150	39.09	80	1613
CR031	JAUQTR000000000	1,653,356	50,390	38.92	58	1601
CR045	JAUQTS000000000	1,687,749	63,377	39.07	127	1593
CR047	JAUQTT000000000	1,686,534	69,457	39.52	75	1644
CR048	JAUQTU000000000	1,692,916	58,084	38.85	73	1649
CR054	JAUQTV000000000	1,595,752	65,233	38.90	74	1561
AP029	JAUQTW000000000	1,617,151	48,931	39.24	310	1796

* Note: The N50 is a statistical measure used in bioinformatics to assess the quality of genome assembly. A higher N50 value indicates a better assembly.

**Table 2:** Description of origin and clinical source of the Colombian genomes analyzed in this study.

Strain Name	Sex	Age	Diagnosis	Municipality
AP002	F ^a^	52	Intestinal Metaplasia	Florida
AP015	M ^b^	50	Intestinal Metaplasia	Florida
AP018	F	47	Intestinal Metaplasia	Florida
AP021	F	50	Intestinal Metaplasia	Florida
AP022	F	60	Non Atrophic Gastritis	Florida
AP025	F	65	Chronic Active Gastritis	Florida
AP028	F	54	Chronic Active Gastritis	Florida
AP031	F	52	Chronic Active Gastritis	Florida
CR004	M	68	Non Atrophic Gastritis	Pasto
CR005	F	54	Non Atrophic Gastritis	Pasto
CR031	F	50	Non Atrophic Gastritis	Pasto
CR045	M	34	Non Atrophic Gastritis	Pasto
CR047	F	62	Non Atrophic Gastritis	Pasto
CR048	F	48	Non Atrophic Gastritis	Pasto
CR054	F	30	Non Atrophic Gastritis	Pasto
AP029	F	48	Intestinal Metaplasia	Pasto

Note: ^a^: Female, ^b^: Male.

**Table 3:** Gene mutations that confer rifampicin and furazolidone resistance in *Helicobacter pylori*.

Antibiotic	Gene	Mutations	Reference
Rifampicin	*rpoB*	V149F, Q527R, D530 and H540	[27,28]
Furazolidone	*porD*	G353A, A356G and C357T	[29]

## Data Availability

The datasets presented in this study can be found in online repositories. The names of the repository/repositories and accession number(s) can be found below: https://pubmlst.org/organisms?title=Helicobacter+pylori (accessed on 8 August 2023).
